# Discovery of a Natural Product-Like iNOS Inhibitor by Molecular Docking with Potential Neuroprotective Effects *In Vivo*

**DOI:** 10.1371/journal.pone.0092905

**Published:** 2014-04-01

**Authors:** Hai-Jing Zhong, Li-Juan Liu, Cheong-Meng Chong, Lihua Lu, Modi Wang, Daniel Shiu-Hin Chan, Philip Wai Hong Chan, Simon Ming-Yuen Lee, Dik-Lung Ma, Chung-Hang Leung

**Affiliations:** 1 State Key Laboratory of Quality Research in Chinese Medicine, Institute of Chinese Medical Sciences, University of Macau, Macao, China; 2 Department of Chemistry, Hong Kong Baptist University, Kowloon Tong, Hong Kong, China; 3 Division of Chemistry and Biological Chemistry, School of Physical and Mathematical Sciences, Nanyang Technological University, Singapore, Singapore; Bioinformatics Institute, Singapore

## Abstract

In this study, we applied structure-based virtual screening techniques to identify natural product or natural product-like inhibitors of iNOS. The iNOS inhibitory activity of the hit compounds was characterized using cellular assays and an *in vivo* zebrafish larvae model. The natural product-like compound **1** inhibited NO production in LPS-stimulated Raw264.7 macrophages, without exerting cytotoxic effects on the cells. Significantly, compound **1** was able to reverse MPTP-induced locomotion deficiency and neurotoxicity in an *in vivo* zebrafish larval model. Hence, compound **1** could be considered as a scaffold for the further development of iNOS inhibitors for potential anti-inflammatory or anti-neurodegenerative applications.

## Introduction

Nitric oxide (NO) is a short-lived pleiotropic regulator that plays a diverse variety of roles in living organisms. NO controls vascular tone and blood flow by inhibiting vascular smooth muscle contraction and growth, platelet aggregation, and leukocyte adhesion [1]. Moreover, NO mediates mitochondrial oxygen consumption through inhibition of cytochrome c oxidase [2].

Endogenous NO is produced from *L*-arginine, oxygen and NADPH, in a series of reactions catalyzed by homodimers of NO synthases (NOS) [3]. There are three isoforms of NOS: endothelial NOS (eNOS), neuronal NOS (nNOS) and inducible NOS (iNOS) [3]. The three NOS isoforms share a common structure that is comprised of two major domains. The N-terminal catalytic domain binds the heme prosthetic group, the substrates *L*-arginine and oxygen, and the redox cofactor 5,6,7,8-tetrahydrobiopterin (H_4_B), and is linked *via* a calmodulin-recognition site to a C-terminal reductase domain that contains binding sites for NADPH, FAD and FMN. Aided by the heme group, electrons are transferred from NADPH to oxygen, *via* the cofactors FAD and FMN [4]. A structural zinc atom exists at the interface region of NOS dimers, and its coordination to two cysteine residues of each subunit has been proposed to stabilize dimer formation [5].

eNOS and nNOS are constitutive enzymes regulated by the levels of Ca^2+^ and calmodulin within the cell [6]. On the other hand, iNOS activity is Ca^2+^-independent, and its expression can be up-regulated in macrophages and other tissues in response to inflammatory signals. Sustained induction of iNOS activity can lead to the enhanced formation of reactive intermediates of NO, which can cause DNA damage, inhibit DNA repair, modify cell signaling, and promote proinflammatory and angiogenic activities of the cell [7]. Furthermore, the overproduction of NO by iNOS in the brain has been implicated in the development of Parkinson’s disease, which is characterized by the slow and progressive degeneration of dopaminergic neurons in the substantia nigra [8].

Nature provides a diverse cornucopia of bioactive substructures and motifs for the medicinal chemist [9]. Historically, natural products have represented an important source of molecular scaffolds for the development of new drugs. For example, Newman and Cragg have shown that of the 175 small molecules approved for the treatment of cancer since the 1940s, 85 (48.6%) of these were either natural products themselves or derived directly from natural products [10]. However, the shift towards high-throughput screening technologies in the pharmaceutical industry over the past few decades has somewhat tempered the enthusiasm for natural product chemistry, whose structures were deemed too complex and whose extracts too dirty to be compatible with the highly automated drug discovery methodologies that were developed [11].

Meanwhile, virtual screening has emerged as an efficient technique for the rapid identification and optimization of potential hit compounds [12]–[17]. Virtual screening allows the remarkable structural diversity and fascinating molecular architecture exhibited by natural products to be harnessed in an efficient and inexpensive manner [18]–[20]. For example, non-binders can be predicted *in silico*, reducing the need to isolate or synthesise large quantities of natural products or their analogies. Furthermore, issues associated with bioassay-guided fractionation and dereplication of natural product extracts can be effectively bypassed. Based on these ideas, we sought to apply structure-based virtual screening techniques to identify natural product or natural product-like inhibitors of iNOS.

## Materials and Methods

### Molecular Docking and Virtual Screening

**Model construction.**

- The initial model of iNOS was built from an X-ray co-crystal structure of the monomeric oxygenase domain of murine Δ114 iNOS complexed with an inhibitor (PDB: 1DD7) [21], using the molecular conversion procedure implemented in the ICM-pro 3.6-1d program (Molsoft) [22].
- Hydrogen and missing heavy atoms were added to the receptor structure, and atom types and partial charges were assigned.
- The model was subjected to local energy minimization to identify the optimal position by using the conjugate gradient algorithm and analytical derivatives in the internal coordinates.

**High throughput molecular docking.**

- Over 90,000 natural product or natural product-like compounds from the ZINC natural product database were docked to the molecular model of iNOS *in silico.* Molecular docking was performed using the virtual library screening (VLS) module in the ICM-Pro 3.6-1d program (Molsoft).
- Each compound in the library was assigned the MMFF3 force field atom types and charges and was then subjected to Cartesian minimization. During the docking analysis, the ligand was considered flexible and the binding pose and internal torsions were sampled by the biased probability Monte Carlo (BPMC) minimization procedure, which involved local energy minimization after each random move.
- Each compound was docked to the protein complex binding pocket, and a score from the docking was assigned to each compound according to the weighed component of the ICM scoring function described below. Each compound was docked three times to ensure the convergence of the Monte Carlo optimization, and the minimum score of each ligand from the three independent docking experiments was retained and used for ranking.

**ICM full-atom ligand-receptor complex refinement and scoring.**

- According to the ICM method [23], the molecular system was described using internal coordinates as variables. Energy calculations were based on the ECEPP/3 force field with a distance-dependent dielectric constant.
- The BPMC minimization procedure was used for global energy optimization. The BPMC global-energy-optimization method consists of 1) a random conformation change of the free variables according to a predefined continuous probability distribution; 2) local-energy minimization of analytical differentiable terms; 3) calculation of the complete energy including nondifferentiable terms such as entropy and solvation energy; 4) acceptance or rejection of the total energy based on the Metropolis criterion and return to step (1).
- The binding between the small molecules and iNOS were evaluated with a full-atom ICM ligand binding score from a multireceptor screening benchmark as a compromise between approximated Gibbs free energy of binding and numerical errors. The score was calculated by: *S*_bind_ = *E*_int_+*T*Δ*S*_Tor_+*E*_vw_+α_1_ *E*_el_+α_2_ *E*_hb_+α_3_ *E*_hp_+α_4_ *E*_sf_, where *E*_vw_, *E*_el_, *E*_hb_, *E*_hp_, and *E*_sf_ are van der Waals, electrostatic, hydrogen bonding, and nonpolar and polar atom solvation energy differences between bound and unbound states, respectively. *E*_int_ is the ligand internal strain, Δ*S*_Tor_ is its conformational entropy loss upon binding, *T* = 300 K, and α_i_ are ligand- and receptor independent constants. The calculated binding score of –31.74 reflects the strong interaction between compound **1** and the iNOS binding site.

### Materials

Griess reagent system was purchased from Promega. LPS was purchased from Merck Millipore. MTT (3-(4,5-dimethylthiazolyl)-2,5-diphenyltetrazolium bromide) was purchased from Sigma-Aldrich (Germany). MPTP (1-methyl-4-phenyl-1,2,3,6-tetrahydropyridine) and L-deprenyl (Selegiline) were purchased from Sigma-Aldrich (Germany).

### Griess Assay

Raw264.7 cells were seeded at a density of 10^6^ cells/well in a 24-well plate, and incubated for 12 h. Compounds or vehicle was added to the cells at the indicated concentrations. After 15 min, LPS (1 μg/mL) was added to each well, and the cells were incubated for a further 18 h. The level of NO production was evaluated using the Griess method.

### MTT Assay

Raw264.7 cells were seeded at a density of 10^5^ cells/well in a 96-well plate and incubated overnight at 37°C. Compounds or vehicle was added to the cells at the indicated concentrations. The cells were incubated for 18 h. MTT (3-(4,5-dimethylthiazolyl)-2,5-diphenyltetrazolium bromide) was added to each well, and the cells were incubated for a further 4 h. DMSO was added to solubilize formazan. The absorbance at λ = 570 nm was measured by a microplate reader. The IC_50_ values of the compounds were determined by the dose dependence of the surviving cells after exposure to the compounds for 16 h.

### Fish Maintenance

Wild-type zebrafish were used in this study. Embryos were collected after natural spawning, staged according to standard criteria, and raised synchronously at 28.5°C in embryo medium (13.7 mM NaCl, 540 μM KCl, pH 7.4, 25 μM Na_2_HPO_4_, 44 μM KH_2_PO_4_, 300 μM CaCl_2_, 100 μM MgSO_4_, 420 μM NaHCO_3_, pH 7.4). No additional maintenance was required as the embryos receive nourishment from the attached yolk. Ethical approval for the animal experiments was granted by the Animal Research Ethics Committee, University of Macau.

### Zebrafish Locomotion Assay

1 dpf zebrafish embryos were co-incubated with 250 μM MPTP and compound **1** or *L*-deprenyl at the indicated concentrations for 3 days. Zebrafish larvae at 4 dpf were transferred into 96-well plates (1 fish/well and 12 larvae/group). Zebrafish behavior was monitored and those fish showing signs of excessive stress reaction to handling (such as rapid and disorganized swimming or immobility for 2 min) were discarded. The experiments were performed in a calm sealed area. The larvae were allowed to habituate to the new environment for 30 min. Behavior was monitored by an automated video tracking system (Viewpoint, ZebraLab, LifeSciences). The 96-well plates and camera were housed inside a Zebrabox and the swimming pattern of each fish was recorded for 10 min. Three 10-minute sessions was recorded for each fish. The total distance moved was defined as the distance (in mm) that the fish had moved during one session (10 min). Statistical analysis of the total distance travelled by each zebrafish larva in the different treatment groups was performed (ANOVA and Dunnett’s test).

### Whole-mount Immunostaining with Antibody against Tyrosine Hydroxylase

Zebrafish at 1 dpf were co-incubated with MPTP (250 μM) and *L*-deprenyl (20 μM) or the indicated concentrations of compound **1** for 48 h. Whole-mount immunostaining in zebrafish was performed as previously described by Zhang *et al.* [24]. Briefly, zebrafish were fixed in 4% paraformaldehyde in PBS for 5 h. Fixed samples were blocked (2% lamb serum and 0.1% BSA in PBST) for 1 h at room temperature. The samples were incubated with mouse monoclonal anti-tyrosine hydroxylase (TH) antibody (Millipore, Billerica, MD, USA) overnight at 4°C. On the next day, samples were washed six times with PBST (each wash lasted 30 min), followed by incubation with Alexa Fluor 488 goat anti-mouse antibody. After staining, zebrafish were flat-mounted with 3.5% methylcellulose and photographed. Semi-quantification of the area of TH^+^ cells was assessed by an investigator, unaware of the drug treatment, using Image-Pro Plus 6.0 software (Media Cybernetics, Silver Spring, MD). Results are expressed as percentage of area of TH^+^ cells in untreated normal control groups. Statistical analysis of TH density was performed with 10 fish in each group. Statistical analysis was performed using ANOVA and Dunnett’s test.

### Analysis of NOS Protein Expression by Western Blotting

The protein expression of nNOS, eNOS and iNOS was examined in HepG2 cells. Cells were treated with the indicated concentrations of SMT and compound **1** (1 or 5 μM) for 12 h. 1×10^6^ HepG2 cells were washed three times with cold PBS, resuspended in RIPA lysis buffer, and incubated on ice for 30 min. The cell debris was then centrifuged at 16,000 rpm for 30 min at 4°C. Sample protein content in the supernatant was determined by spectrophotometric assay (BCA Protein Assay Kit, Pierce, IL, USA). Equal protein amounts were electrophoresed on an 8% SDS-PAGE and transferred to a PVDF membrane. The membranes were incubated in 5% milk at room temperature for 1 h. After blocking of nonspecific binding sites, the membranes were incubated at 4°C overnight in 5% milk containing one of the following antibodies: rabbit polyclonal antibody against nNOS (diluted 1∶500, Cell Signaling Technology), eNOS (diluted 1∶500, Cell Signaling Technology) or iNOS (diluted 1∶500, Cell Signaling Technology). After washing with Tween-PBS (PBST), the membranes were incubated in 5% milk containing a goat-antirabbit IgG-HRP (sc-2030, Santa Cruz Biotechnology) at room temperature for 2 h. After washing with PBST, expressions of NOS were detected by enhanced chemiluminescence (ECL).

## Results

### High-throughput Virtual Screening of Natural Product or Natural Product-like Library

**Figure 1:**
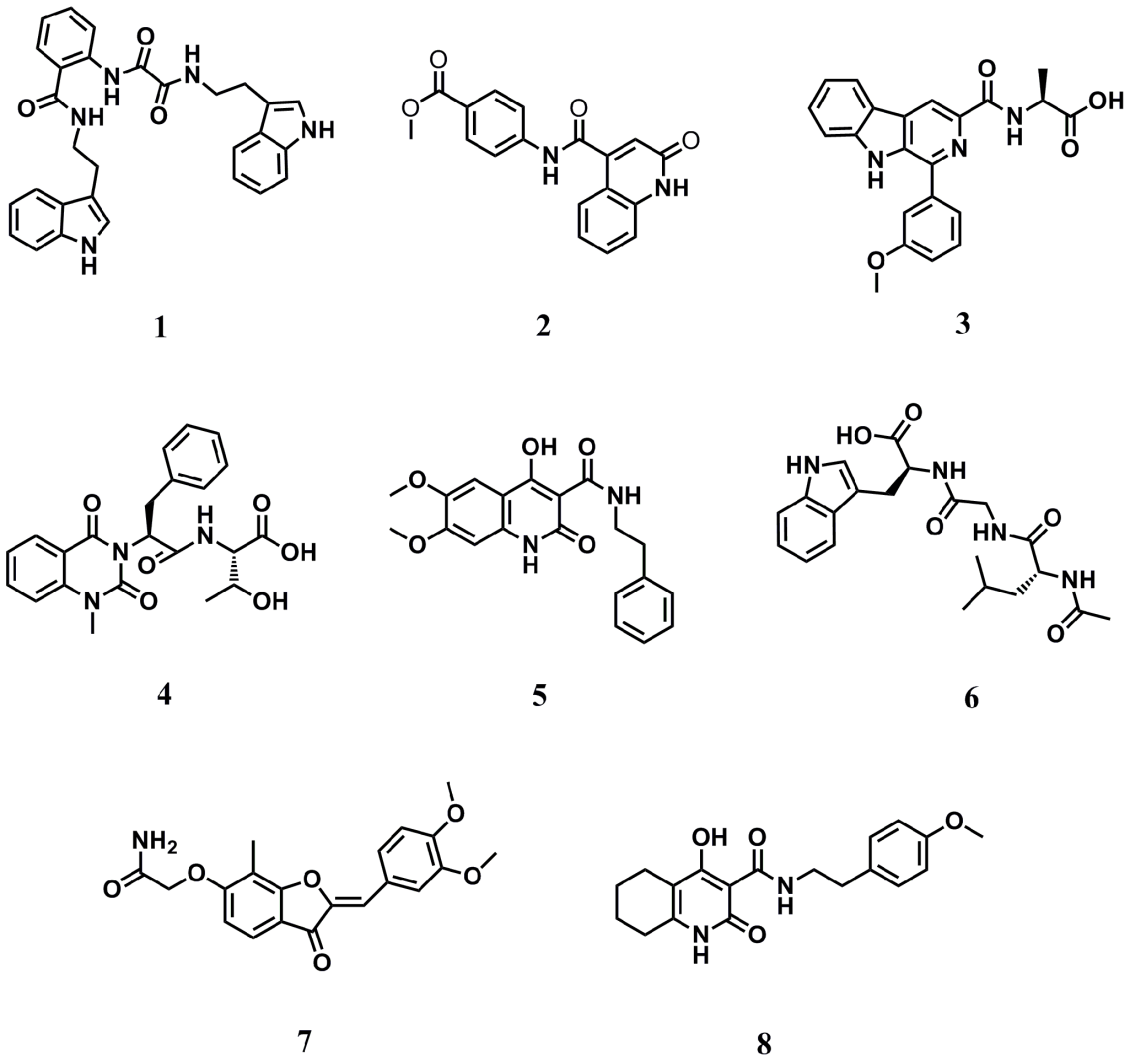
Structures of highest-scoring 8 compounds identified in the high-throughput virtual screening chosen for biological validation.

We constructed the initial model of iNOS from the X-ray co-crystal structure of the monomeric oxygenase domain of murine Δ114 iNOS complexed with the inhibitor *N*-[(1,3-benzodioxol-5-yl)methyl]-1-[2-(1*H*-imidazol-1-yl)pyrimidin-4-yl]-4-(methoxycarbonyl)-piperazine-2-acetamide (the “acetamide”) (PDB: 1DD7) [21] using ICM-pro 3.6–1d (Molsoft) [25]. A chemical library containing over 90,000 natural product or natural product-like compounds (ZINC natural product database) was then docked to the molecular model of iNOS using the Internal Coordinate Mechanics (ICM) method (Molsoft) [22]. The top eight highest-scoring compounds (Figure 1) were obtained based on their availability and accessibility from commercial suppliers. The binding scores of the top eight compounds ranged from –41.595 to –29.786, and their binding scores and docking diagrams are displayed. (Table 1 and Figure S1).

**Table 1:** ICM docking score of the compounds selected for biological screening.

Compound number	Binding score
**1**	−31.742
**2**	−37.227
**3**	−41.251
**4**	−41.595
**5**	−29.786
**6**	−39.522
**7**	−41.264
**8**	−38.490

### Compound 1 was the most Effective at Inhibiting NO Production in LPS-stimulated Macrophages

**Figure 2:**
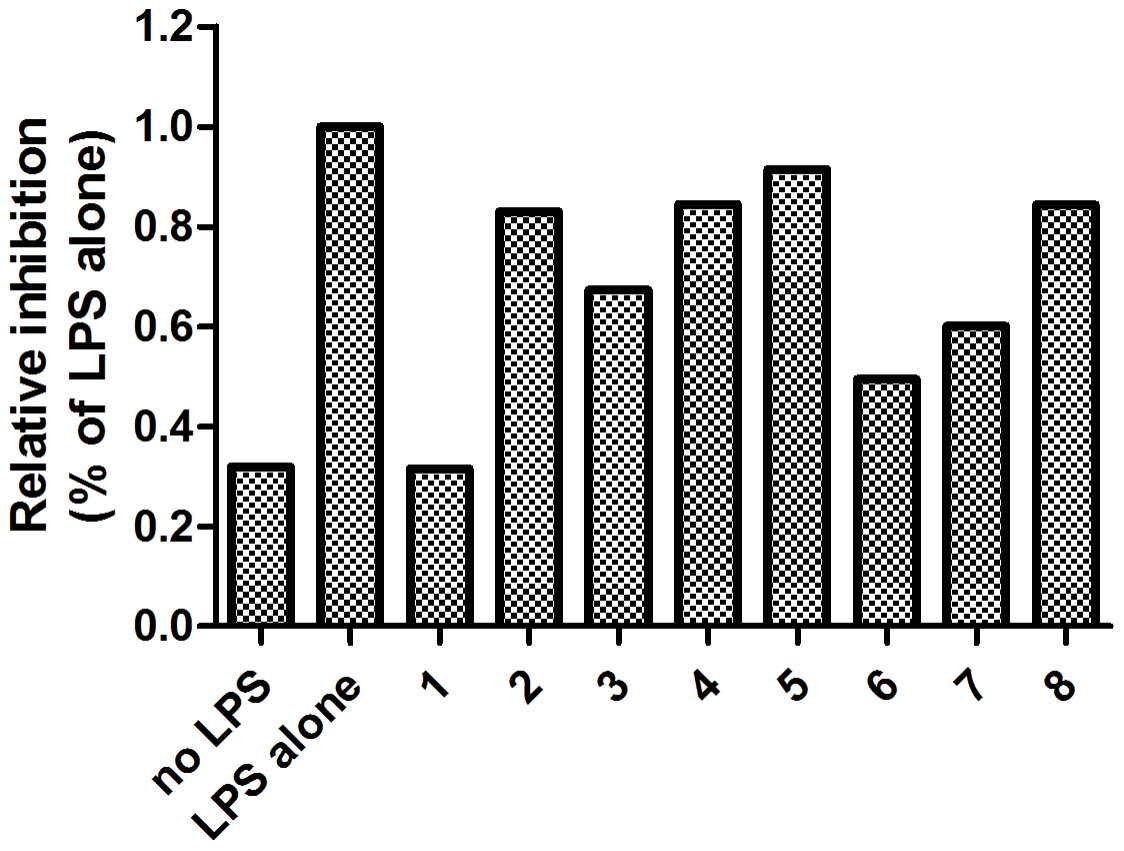
Preliminary assay to evaluate inhibition of NO production in Raw264.7 cells by the hit compounds. Cells were incubated with compounds (20 μM) or vehicle for 15 min, and stimulated with LPS (1 μg/mL). NO production was estimated using the Griess reagent.

The ability of the candidate compounds to inhibit iNOS was evaluated in a preliminary cellular assay (Figure 2). Raw264.7 macrophages were incubated with compounds (20 μM) or vehicle, and stimulated with lipopolysaccharide (LPS), which triggers the production of NO by iNOS [26]. The levels of cellular NO production by iNOS in the cells was evaluated using the Griess method. The results of the preliminary assay showed that compound **1** was the most effective at inhibiting NO production in LPS-stimulated macrophages, with NO release being reduced to 32% of the negative control value at 20 μM of the compound. Significantly, the level of NO release in stimulated macrophages in the presence of 20 μM of compound **1** was approximately equal to that in the unstimulated cells.

### Molecular Modelling Analysis

**Figure 3:**
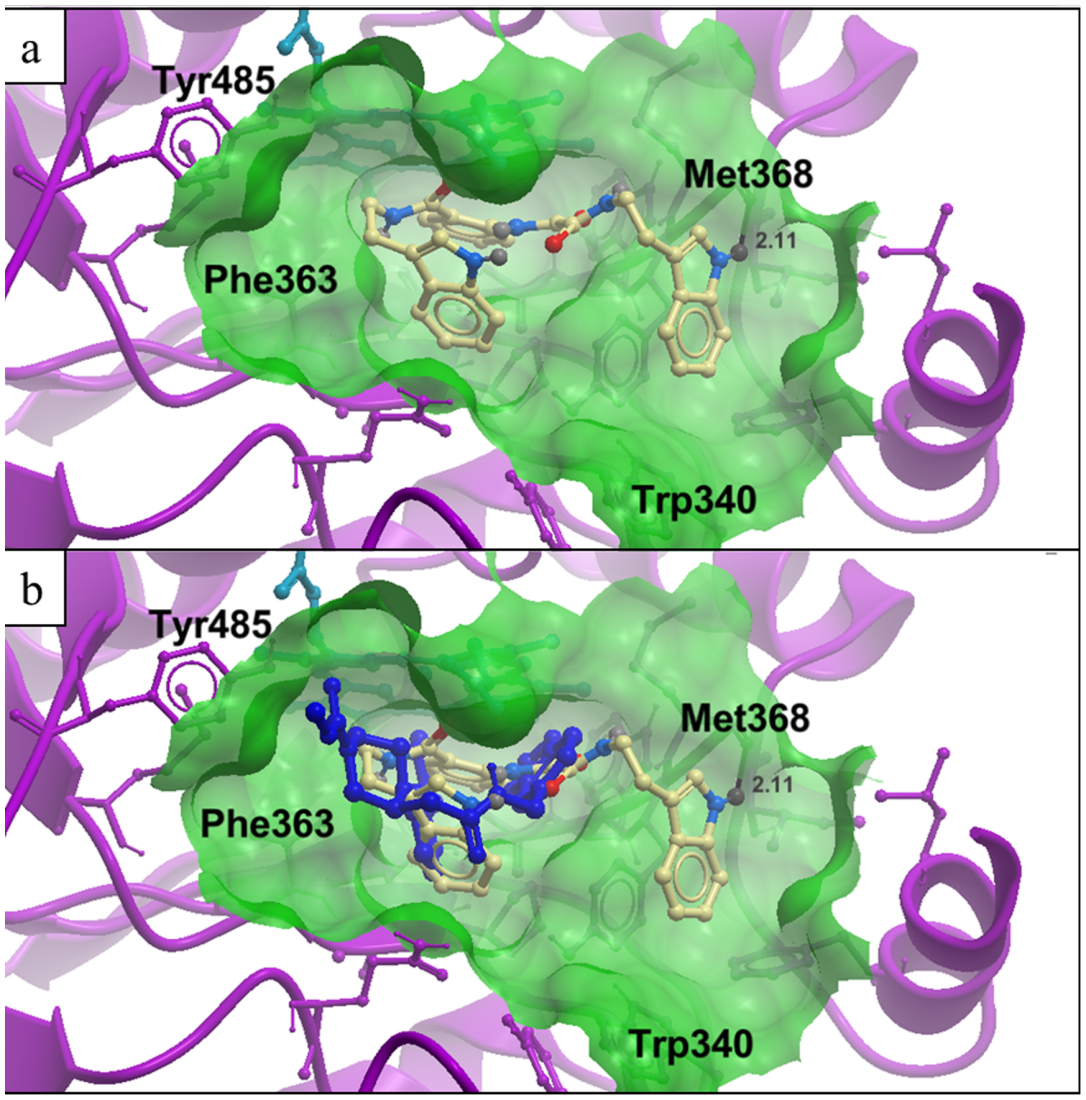
Low-energy binding conformations of compound 1 and the acetamide bound to the iNOS molecular model by computational ligand docking. (a) iNOS is depicted in ribbon form. The heme group and compound **1** are depicted as ball-and-stick models. (b) A superposition of compound **1** and the acetamide, shown as ball-and-stick models.

Molecular modeling was used to investigate the binding mode of compound **1** to iNOS. In the best-scoring binding pose of compound **1** to the active site of iNOS, the NH group of one of the tryptamine units forms a hydrogen bond with the backbone carbonyl group of Met368 (Figure 3A). Furthermore, the tryptamine and phenyl rings of compound **1** are located close to the heme prosthetic group and the aromatic side chains of Tyr341, Phe363 and Tyr485, allowing for possible Van der Waals or even π-stacking interactions with those groups. The superposition of compound **1** and the acetamide bound to iNOS as predicted by molecular docking (Figure 3B) shows that the molecules occupy a similar region of the iNOS binding site.

### Compound 1 Inhibited NO Release in Macrophages with Dose-dependent Manner

**Figure 4:**
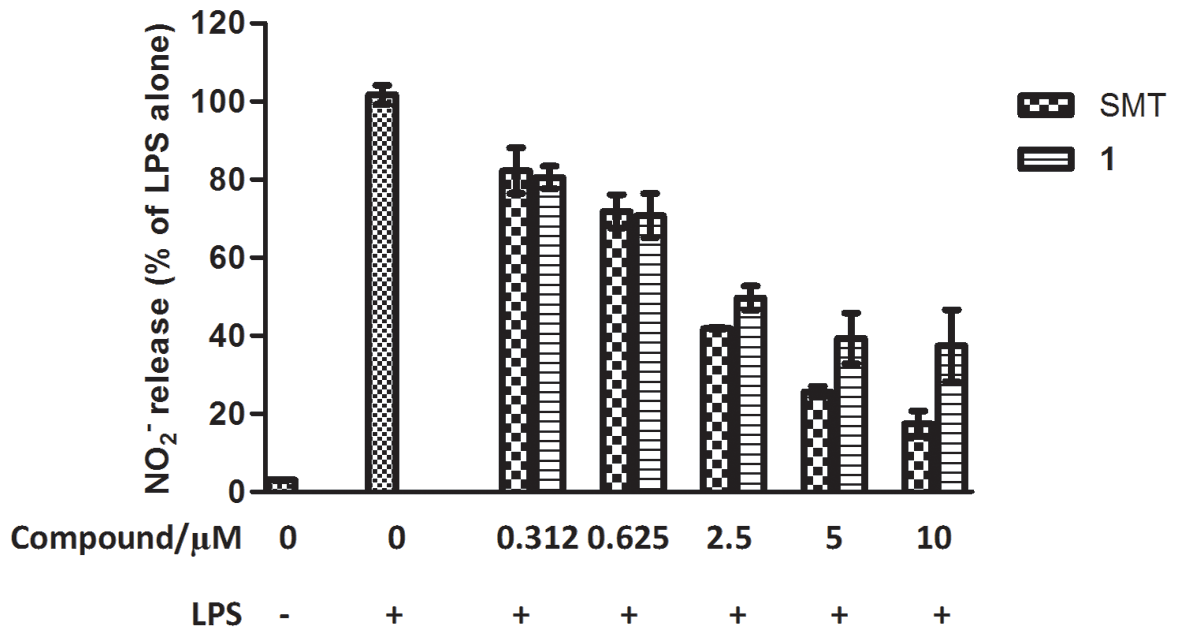
Dose-dependent inhibition of NO production in Raw264.7 cells. Cells were incubated with the indicated concentration of compounds or vehicle for 15(1 μg/mL). NO production was estimated using the Griess reagent. Estimated IC_50_ values for compound **1**∶2.47 μM; SMT: 2.06 μM.

A dose-response experiment was performed to investigate the potency of compound **1** at inhibiting NO release in macrophages (Figure 4). The results revealed that as the concentration of compound **1** was increased, the level of NO production in Raw264.7 cells diminished (IC_50_ = 2.47 μM). Furthermore, the potency of compound **1** was comparable to the positive control *S*-methylisothiourea sulfate (SMT) (IC_50_ = 2.06 μM) in a side-by-side comparison. We reason that the ability of compound **1** to block LPS-induced NO production in macrophages could be likely attributed to the inhibition of iNOS activity by compound **1** *in cellulo*.

### Compound 1 is Relatively Non-cytotoxic

**Figure 5:**
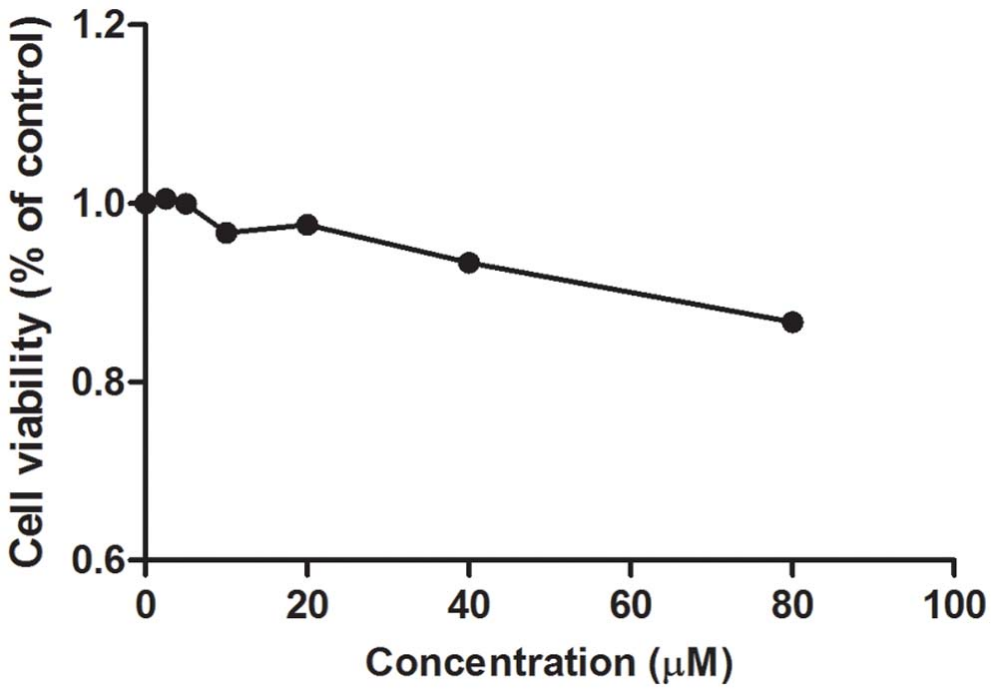
MTT assay to evaluate the cytotoxicity of the compounds towards Raw264.7 cells. Cells were incubated with the indicated concentrations of compounds for 18λ = 570 nm after the addition of MTT. Data are expressed as the percentage of survival of cells compared to the negative control.

We next performed the MTT assay to evaluate the cytotoxicity of compound **1** towards Raw264.7 cells (Figure 5). The IC_50_ value of compound **1** against Raw264.7 cells was estimated to be >80 μM, as growth was only reduced by 14% at the highest concentration of compound **1** (80 μM) tested. This result revealed that compound **1** is relatively non-toxic towards cells.

### The Effect of Compound 1 on the Expression of NOS Isoforms

**Figure 6:**
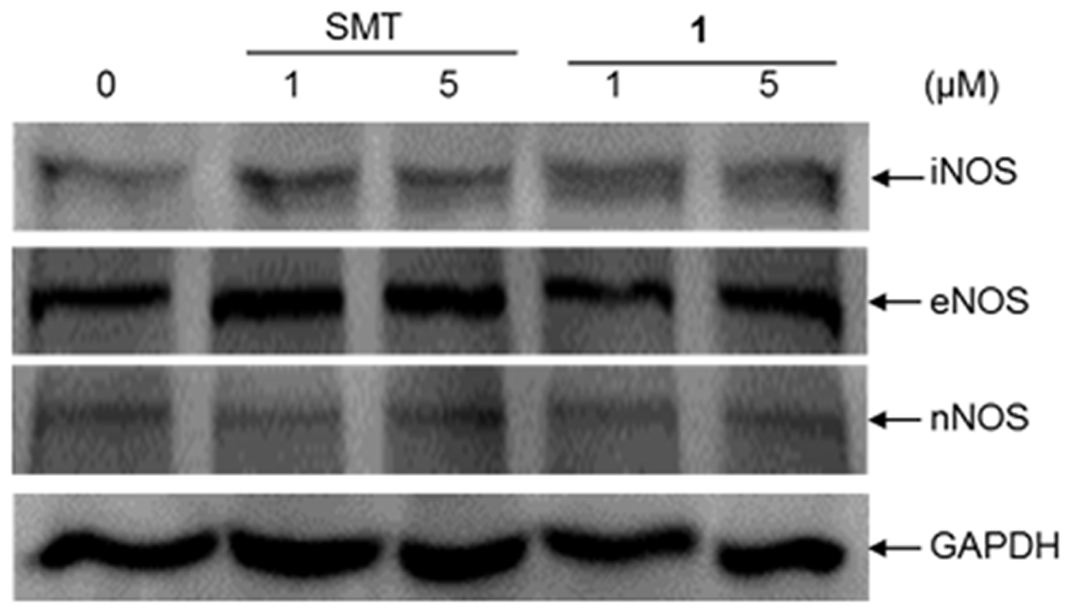
Protein expression of iNOS, eNOS and nNOS in HepG2 cells. HepG2 cells were treated with SMT and compound **1** (1 or 5 μM) or DMSO (vehicle control) for 12 h analysed by Western blotting. Equal protein loading was confirmed by GAPDH content.

The effect of compound **1** on the expression of NOS isoforms in HepG2 cells was examined by Western blotting. The results showed no change in iNOS, eNOS or nNOS expression in cells treated with compound **1** or SMT (up to 5 μM) for 12 h (Figure 6). This suggests that the iNOS inhibitory effects of compound **1** *in cellulo* are not mediated by a decreased in the level of NOS isoforms in the treated cells.

### Compound 1 Reversed MPTP-induced Locomotor Deficiency

**Figure 7:**
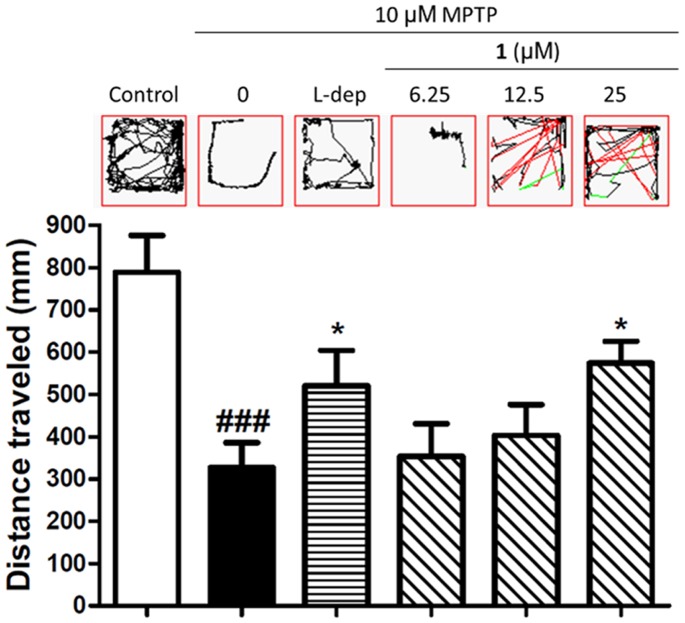
Compound 1 partially reverses MPTP-induced locomotion deficiency in zebrafish larvae. Zebrafish embryos at 1-incubated with MPTP (250 μM) and *L*-deprenyl (20 μM) or the indicated concentrations of compound **1** for 3 d. Each treatment group contained 12 fish larvae, and three independent trials were performed for each fish. The results represented the mean distance travelled by the larvae in 10 min and values are expressed as mean ± SEM. ^###^ *p*<0.001 *vs.* negative control and **p<0.01 *vs.* MPTP.

Zebrafish embryos at 1 dpf were co-incubated with 250 μM of the neurotoxin MPTP (1-methyl-4-phenyl-1,2,3,6-tetrahydropyridine) and the indicated concentrations of compound **1** for 3 days (Figure 7). The monoamine oxidase B inhibitor *L*-deprenyl, which protects against MPTP-induced neurodegeneration *in vivo* [27], was used as a positive control. The results showed that the administration of MPTP to zebrafish larvae resulted in a 59% decrease in locomotor activity. Co-incubation of the larvae with 20 μM of *L*-deprenyl partially reversed MPTP-induced locomotor deficiency, with the total distance travelled over 10 min reduced by 34% compared to the negative control. Encouragingly, compound **1** exhibited a dose-dependent attenuation of MPTP-induced locomotor deficiency in the zebrafish larvae. At 25 μM of compound **1**, the locomotor activity of the fish was reduced by only 27%. These data suggest that compound **1** was able to partially reverse locomotor deficiency induced by MPTP in a zebrafish model.

### Compound 1 Attenuated MPTP-induced Neurotoxicity, with TH^+^ Density Reduced

**Figure 8:**
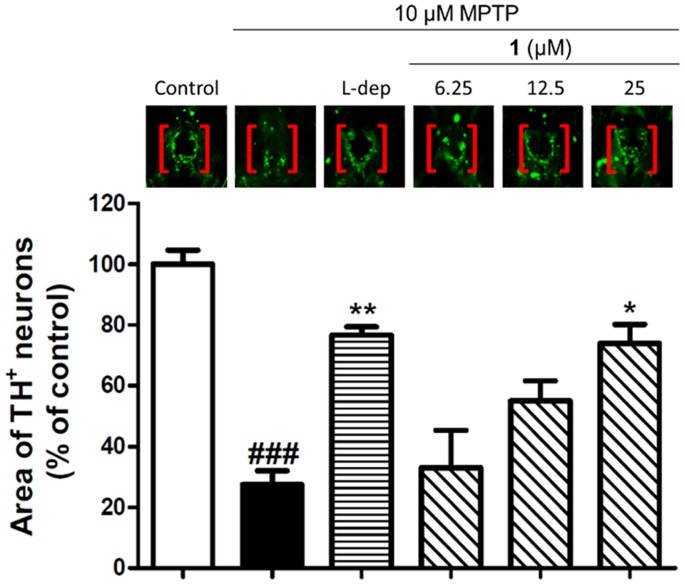
The effect of iNOS inhibitor on MPTP-induced dopaminergic neuron loss in zebrafish. Zebrafish at 1-incubated with MPTP (250 μM) and *L*-deprenyl (20 μM) or the indicated concentrations of compound **1** for 48 h. The morphology of dopaminergic neurons in the zebrafish brain was visualized by immunostaining with anti-tyrosine hydroxylase (TH) antibody. Statistical analysis of TH density was performed with 10 fish in each group. Data are expressed as a percentage of the control group, and values are expressed as mean ± SEM. ^###^ *p*<0.001 *vs.* negative control, *p<0.05 and **p<0.01 *vs.* MPTP.

To further investigate the neuroprotective effect of compound **1** in zebrafish, we visualized the morphology of dopaminergic neurones in the larval brains by immunostaining of tyrosine hydroxylase (TH) (Figure 8). TH activity is important for dopamine synthesis in the brain, and dysregulation of TH activity contributes to Parkinson’s disease [28]. Zebrafish treated with MPTP showed a 72% reduction in TH^+^ immunostaining compared to the untreated control group. Administration of larvae with 20 μM of *L*-deprenyl partially attenuated MPTP-induced neurotoxicity, with TH^+^ density reduced by 23% compared to the negative control. Significantly, compound **1** displayed a dose-dependent alleviation of MPTP-induced neurotoxicity, with TH^+^ staining reduced by only 26% at a concentration of 25 μM. We propose that the neuroprotective effect of compound **1** in zebrafish larvae may be attributed, at least in part, to the inhibition of iNOS by compound **1** *in vivo*, resulting in the protection of dopaminergic neurons in the brain and the attenuation of MPTP-induced locomotor deficiency.

## Discussion

**Figure 9:**
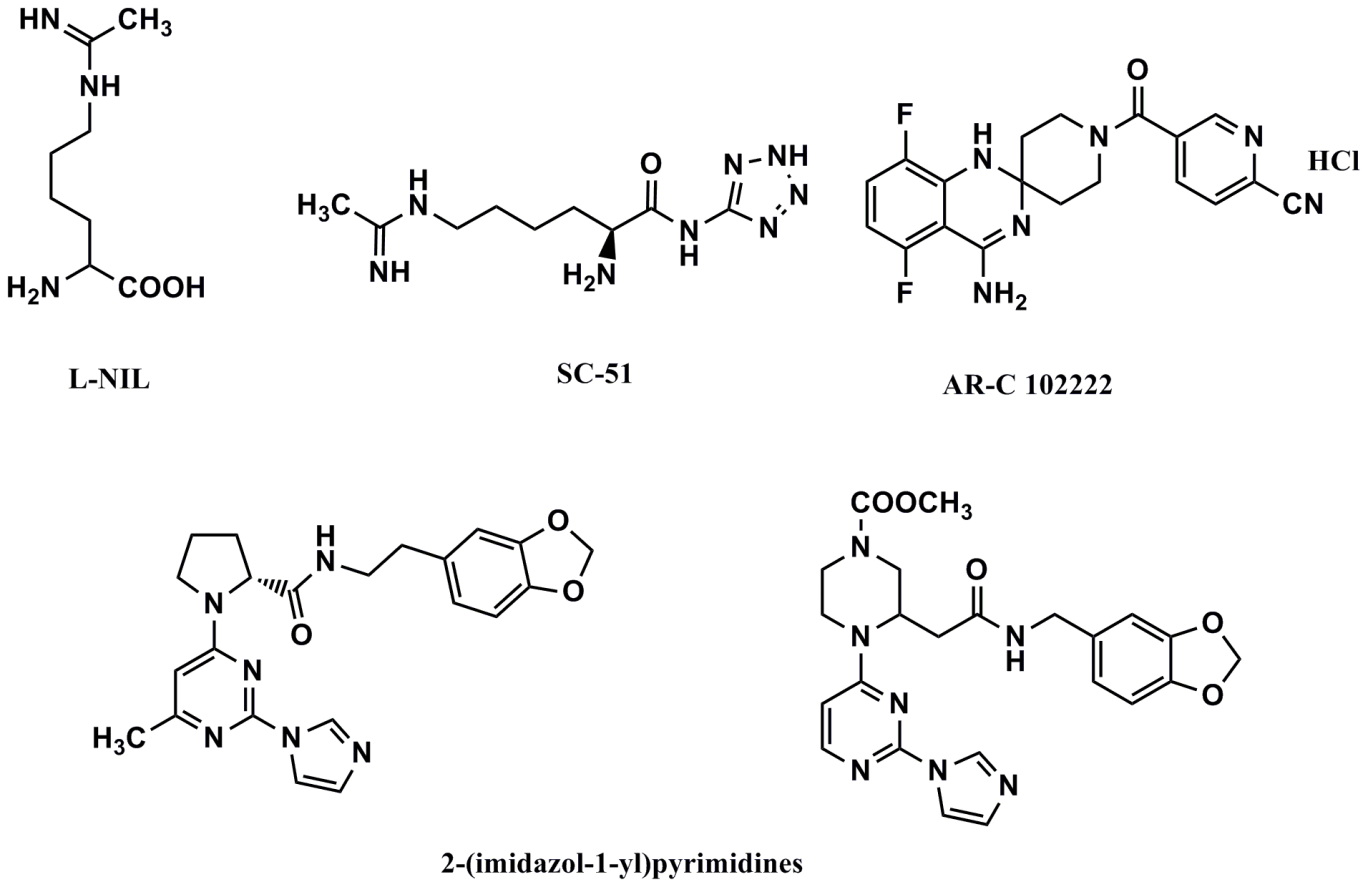
iNOS inhibitors have been reported in the literature.

NO is generated by phagocytes as part of the human immune response, and contributes to the cytotoxic activity of macrophages [29]. However, excessive NO production can lead to numerous pathological processes, such as macrophage-mediated immunity, cancer and neurodegenerative diseases [30]. In particular, the overproduction of NO by the sustained induction of iNOS activity in the brain can lead to Parkinson’s disease [8]. Hence, this has stimulated the development of a variety of iNOS inhibitors (Figure 9). *L-*NIL [31], [32] and its prodrug SC-51 [33] act as mimics of the natural substrate *L*-arginine. Additionally, the quinazolinamine AR-C102222 displayed strong efficacy in an adjuvant-induced rat arthritis model [34]. Inhibitors targeting iNOS dimerization, such as 2-(imidazol-1-yl)pyrimidines [21], [35], have also been discovered through combinatorial chemistry.

The combination of virtual screening and natural products represents a powerful synergy for the cost-effective identification of bioactive molecules [18]–[20]. Therefore, we applied a molecular docking approach to identify inhibitors of iNOS from a database of over 90,000 natural products and natural product-like compounds, using a molecular model constructed from the X-ray co-crystal structure of the monomeric oxygenase domain of murine Δ114 iNOS complexed with a small molecule inhibitor (PDB: 1DD7 [21]). From the high-throughput virtual screening campaign, the eight highest-scoring compounds were purchased based on their commercial availability. A preliminary assay revealed that compound **1** was the most effective at inhibiting NO production in LPS-stimulated macrophages.

The structure of compound **1** contains two tryptamine moieties linked by a phenyloxalamide-type bridge. In mammals, endogenous tryptamines such as serotonin and melatonin were initially characterized as neuromodulatory or neurotransmitter agents in the brain [36]. Some endogenous tryptamines exhibit anti-oxidant and anti-inflammatory activities [37]. The synthetic anti-convulsive and anti-nociceptive *N*-salicyloyltryptamine (STP), which also contains the benzoyltryptamine fragment found in compound **1**, was shown to reverse several redox and inflammatory parameters induced by LPS in Raw264.7 cells [38], though it had no effect on nitrite accumulation. A close analogue of compound **1**, containing an amide linkage instead of an oxalamide bridge, was reported as an antagonist of the CCK1 receptor [39]. To our knowledge, no biological activity of compound **1** has been reported in the literature.

The binding of the acetamide to the iNOS monomer, occupying part of the *L*-arginine binding site, was hypothesized by Devlin *et al.* to allosterically disrupt protein-protein interactions between the monomer units, leading to inhibition of dimerization [21]. Given that compound **1** is predicted to occupy a comparable region of the iNOS binding site compared to the acetamide (Figure 3B), this may be a possible mechanism of action by which compound **1** exerts its anti-iNOS effects. In addition, one of the tryptamine units of compound **1** is located snugly in a small hydrophobic pocket formed by the heme group, the side chain of Phe363 and the side chain of Val346, which is a binding interaction that is observed for a number of iNOS ligands [40], [41]. Furthermore, the ability of the phenyl ring of compound **1** to form possible π-stacking interactions with the heme group may be an important feature contributing to its binding potency, as hypothesized for a series of selective iNOS inhibitors reported by Cheshire *et al.* [42]. The NH group was compound **1** was also predicted to form a hydrogen bond with the backbone carbonyl group of Met368. By comparison, a reversed hydrogen bonding interaction involving Met368 was observed in the X-ray crystal structure of the known iNOS inhibitor 3-bromo-7-nitroindazole with iNOS, where the nitro group of the molecule interacts with the amide group of Met368 [43].

In a dose-response experiment, the level of NO production in stimulated Raw264.7 macrophages decreased as the concentration of compound **1** was increased, confirming that compound **1** is able to block LPS-induced NO production in living cells. Furthermore, an MTT assay revealed that compound **1** is relatively non-toxic towards Raw264.7 cells. Importantly, this data showed that the anti-iNOS activity of compound **1** could achieve at benign concentrations of the compound. Compound **1** also had no effect on iNOS, eNOS or nNOS expression in HepG2 cells, suggesting that its iNOS inhibitory activity is not mediated by a decreased in NOS expression.

The neurotoxin MPTP (1-methyl-4-phenyl-1,2,3,6-tetrahydropyridine) up-regulates iNOS activity, leading to dopaminergic neurodegeneration in a murine model of Parkinson’s disease [44]. iNOS-deficient mice were almost completely protected from MPTP-induced toxicity [45]. Treatment of adult or larval zebrafish with MPTP resulted in pronounced reduction in locomotor activity [46], [47]. Rubenstein *et al.* have demonstrated that the toxicity of MPTP on dopaminergic neurons in zebrafish larvae is mediated by the same pathways that have been characterized in mammals [48]. Therefore, we were interested to determine whether compound **1** could protect against MPTP-induced neurotoxicity in zebrafish larvae.

In the *in vivo* zebrafish larvae model, compound **1** partially reversed locomotor deficiency induced by MPTP in a dose-dependent manner. Furthermore, compound **1** could block MPTP-induced neurotoxicity, as revealed by a reduction in TH^+^ staining. Notably, compound **1** displayed comparable potency to the positive control compound *L*-deprenyl. These results suggest that compound **1** can protect against dopaminergic neurodegeneration induced by MPTP in the brain, which could be attributed, at least partially, to the inhibition of iNOS by compound **1** *in vivo*.

In conclusion, we have identified a natural product-based inhibitor of iNOS by structure-based virtual screening. Compound **1** inhibited NO release in Raw264.7 macrophages stimulated by LPS, and partially reversed MPTP-induced locomotor deficiency and neurotoxicity in a zebrafish larval model. These data suggest that compound **1** could be considered as a promising scaffold for the further development of iNOS inhibitors for potential anti-inflammatory or anti-neurodegenerative applications.

## Acknowledgments

We thank Zhong Ai for his helpful comments.

## Supporting Information

Figure S1 **Low-energy binding conformations of compound (a**–**h) 1–8 bound to the iNOS molecular model by computational ligand docking.** iNOS is depicted in ribbon form. The heme group and compound **1–8** are depicted as ball-and-stick models.
(ZIP)

## References

[1] Luiking YC, Engelen MP, Deutz NE (2010) Regulation of nitric oxide production in health and disease. Curr Opin Clin Nutr Metab Care 13: 97–104. 19841582 10.1097/MCO.0b013e328332f99d PMC2953417

[2] Brown GC (2001) Regulation of mitochondrial respiration by nitric oxide inhibition of cytochrome c oxidase. Biochim Biophys Acta 1504: 46–57. 11239484 10.1016/s0005-2728(00)00238-3

[3] Moncada S, Higgs A (1993) The L-arginine-nitric oxide pathway. N Engl J Med 329: 2002–2012. 7504210 10.1056/NEJM199312303292706

[4] Alderton WK, Cooper CE, Knowles RG (2001) Nitric oxide synthases: structure, function and inhibition. Biochem J 357: 593–615. 11463332 10.1042/0264-6021:3570593 PMC1221991

[5] Hemmens B, Goessler W, Schmidt K, Mayer B (2000) Role of bound zinc in dimer stabilization but not enzyme activity of neuronal nitric-oxide synthase. J Biol Chem 275: 35786–35791. 10954720 10.1074/jbc.M005976200

[6] Forstermann U, Boissel JP, Kleinert H (1998) Expressional control of the ‘constitutive’ isoforms of nitric oxide synthase (NOS I and NOS III). FASEB J 12: 773–790. 9657518

[7] Aktan F (2004) iNOS-mediated nitric oxide production and its regulation. Life Sci 75: 639–653. 15172174 10.1016/j.lfs.2003.10.042

[8] Hirsch EC, Hunot S (2009) Neuroinflammation in Parkinson’s disease: a target for neuroprotection? Lancet Neurol 8: 382–397. 19296921 10.1016/S1474-4422(09)70062-6

[9] Butler MS (2004) The role of natural product chemistry in drug discovery. J Nat Prod 67: 2141–2153. 15620274 10.1021/np040106y

[10] Newman DJ, Cragg GM (2012) Natural Products As Sources of New Drugs over the 30 Years from 1981 to 2010. J Nat Prod 75: 311–335. 22316239 10.1021/np200906s PMC3721181

[11] Li JWH, Vederas JC (2009) Drug Discovery and Natural Products: End of an Era or an Endless Frontier? Science 325: 161–165. 19589993 10.1126/science.1168243

[12] Schneider G (2010) Virtual screening: an endless staircase? Nat Rev Drug Discov 9: 273–276. 20357802 10.1038/nrd3139

[13] Lee H-M, Chan D-S, Yang F, Lam H-Y, Yan S-C, et al (2010) Identification of natural product fonsecin B as a stabilizing ligand of c-myc G-quadruplex DNA by high-throughput virtual screening. Chem Commun 46: 4680–4682. 10.1039/b926359d 20383387

[14] Leung C-H, Chan DS-H, Yang H, Abagyan R, Lee SM-Y, et al (2010) Structure-based discovery of natural-product-like TNF-alpha inhibitors. Angew Chem Int Ed Engl 49: 2860–2864. 20235259 10.1002/anie.200907360 PMC4162403

[15] Leung C-H, Chan DS-H, Hui Yang RA, Lee SM-Y, Zhu G-Y, et al (2011) A natural product-like inhibitor of NEDD8-activating enzyme. Chem Commun 47: 2511–2513. 10.1039/c0cc04927a 21240405

[16] Zhong H-J, Ma VP-Y, Cheng Z, Chan DS-H, He H-Z, et al (2012) Discovery of a natural product inhibitor targeting protein neddylation by structure-based virtual screening. Biochimie 94: 2457–2460. 22709868 10.1016/j.biochi.2012.06.004

[17] Tanrikulu Y, Kruger B, Proschak E (2013) The holistic integration of virtual screening in drug discovery. Drug Discov Today 18: 358–364. 23340112 10.1016/j.drudis.2013.01.007

[18] Ma D-L, Chan D-H, Leung C-H (2011) Molecular docking for virtual screening of natural product databases. Chem Sci 2: 1656–1665.

[19] Rollinger JM, Stuppner H, Langer T (2008) Virtual screening for the discovery of bioactive natural products. In: Petersen F and A R, editors. Natural Compounds as Drugs. Basel, Switzerland: Birkhäuser Verlag. 211–249. 10.1007/978-3-7643-8117-2_6 PMC7124045 18084917

[20] Schuster D, Wolber G (2010) Identification of Bioactive Natural Products by Pharmacophore-Based Virtual Screening. Curr Pharm Des 16: 1666–1681. 20222852 10.2174/138161210791164072

[21] McMillan K, Adler M, Auld DS, Baldwin JJ, Blasko E, et al (2000) Allosteric inhibitors of inducible nitric oxide synthase dimerization discovered via combinatorial chemistry. Proc Natl Acad Sci U S A 97: 1506–1511. 10677491 10.1073/pnas.97.4.1506 PMC26464

[22] Totrov M, Abagyan R (1997) Flexible protein-ligand docking by global energy optimization in internal coordinates. Proteins Suppl 1: 215–220. 10.1002/(sici)1097-0134(1997)1+<215::aid-prot29>3.3.co;2-i 9485515

[23] Schapira M, Totrov M, Abagyan R (1999) Prediction of the binding energy for small molecules, peptides and proteins. J Mol Recognit 12: 177–190. 10398408 10.1002/(SICI)1099-1352(199905/06)12:3<177::AID-JMR451>3.0.CO;2-Z

[24] Zhang ZJ, Cheang LC, Wang MW, Lee SM (2011) Quercetin exerts a neuroprotective effect through inhibition of the iNOS/NO system and pro-inflammation gene expression in PC12 cells and in zebrafish. Int J Mol Med 27: 195–203. 21132259 10.3892/ijmm.2010.571

[25] Abagyan AR, Rausch E, Budagyan L, Totrov M (2009) ICM Manual, 3.0, MolSoft LLC, La Jolla, CA.

[26] Arias-Salvatierra D, Silbergeld EK, Acosta-Saavedra LC, Calderon-Aranda ES (2011) Role of nitric oxide produced by iNOS through NF-κB pathway in migration of cerebellar granule neurons induced by Lipopolysaccharide. Cell Signal 23: 425–435. 20955790 10.1016/j.cellsig.2010.10.017

[27] Ando K, Maeda J, Inaji M, Okauchi T, Obayashi S, et al (2008) Neurobehavioral protection by single dose l-deprenyl against MPTP-induced parkinsonism in common marmosets. Psychopharmacology (Berl) 195: 509–516. 17879087 10.1007/s00213-007-0929-2

[28] Zhu Y, Zhang J, Zeng Y (2012) Overview of tyrosine hydroxylase in Parkinson’s disease. CNS Neurol Disord Drug Targets 11: 350–358. 22483316 10.2174/187152712800792901

[29] Coleman JW (2001) Nitric oxide in immunity and inflammation. Int Immunopharmacol 1: 1397–1406. 11515807 10.1016/s1567-5769(01)00086-8

[30] Pacher P, Beckman JS, Liaudet L (2007) Nitric oxide and peroxynitrite in health and disease. Physiol Rev 87: 315–424. 17237348 10.1152/physrev.00029.2006 PMC2248324

[31] Moore WM, Webber RK, Jerome GM, Tjoeng FS, Misko TP, et al (1994) L-N6-(1-iminoethyl)lysine: a selective inhibitor of inducible nitric oxide synthase. J Med Chem 37: 3886–3888. 7525961 10.1021/jm00049a007

[32] Connor JR, Manning PT, Settle SL, Moore WM, Jerome GM, et al (1995) Suppression of adjuvant-induced arthritis by selective inhibition of inducible nitric oxide synthase. Eur J Pharmacol 273: 15–24. 7537678 10.1016/0014-2999(94)00672-t

[33] Hansel TT, Kharitonov SA, Donnelly LE, Erin EM, Currie MG, et al (2003) A selective inhibitor of inducible nitric oxide synthase inhibits exhaled breath nitric oxide in healthy volunteers and asthmatics. FASEB J 17: 1298–1300. 12738811 10.1096/fj.02-0633fje

[34] Tinker AC, Beaton HG, Boughton-Smith N, Cook TR, Cooper SL, et al (2003) 1,2-Dihydro-4-quinazolinamines: potent, highly selective inhibitors of inducible nitric oxide synthase which show antiinflammatory activity in vivo. J Med Chem 46: 913–916. 12620067 10.1021/jm0255926

[35] Davey DD, Adler M, Arnaiz D, Eagen K, Erickson S, et al (2007) Design, synthesis, and activity of 2-imidazol-1-ylpyrimidine derived inducible nitric oxide synthase dimerization inhibitors. J Med Chem 50: 1146–1157. 17315988 10.1021/jm061319i

[36] Jones RS (1982) Tryptamine: a neuromodulator or neurotransmitter in mammalian brain? Prog Neurobiol 19: 117–139. 6131482 10.1016/0301-0082(82)90023-5

[37] Nosal R, Perecko T, Jancinova V, Drabikova K, Harmatha J, et al (2010) Suppression of oxidative burst in human neutrophils with the naturally occurring serotonin derivative isomer from Leuzea carthamoides. Neuro Endocrinol Lett 31 Suppl 269–72. 21187819

[38] Gasparotto J, de Bittencourt Pasquali MA, Somensi N, Vasques LM, Moreira JC, et al (2013) Effect of N-salicyloyltryptamine (STP), a novel tryptamine analogue, on parameters of cell viability, oxidative stress, and immunomodulation in RAW 264.7 macrophages. Cell Biol Toxicol 29: 175–187. 23605514 10.1007/s10565-013-9245-2

[39] Lassiani L, Pavan MV, Berti F, Kokotos G, Markidis T, et al (2009) Anthranilic acid based CCK1 receptor antagonists: blocking the receptor with the same ‘words’ of the endogenous ligand. Bioorg Med Chem 17: 2336–2350. 19261479 10.1016/j.bmc.2009.02.012

[40] Gradler U, Fuchss T, Ulrich WR, Boer R, Strub A, et al (2011) Novel nanomolar imidazo[4,5-b]pyridines as selective nitric oxide synthase (iNOS) inhibitors: SAR and structural insights. Bioorg Med Chem Lett 21: 4228–4232. 21684157 10.1016/j.bmcl.2011.05.073

[41] Suaifan GARY, Shehadehh M, Al-Ijel H, Taha MO (2012) Extensive ligand-based modeling and in silico screening reveal nanomolar inducible nitric oxide synthase (iNOS) inhibitors. J Mol Graph Model 37: 1–26. 22609742 10.1016/j.jmgm.2012.04.001

[42] Cheshire DR, Aberg A, Andersson GM, Andrews G, Beaton HG, et al (2011) The discovery of novel, potent and highly selective inhibitors of inducible nitric oxide synthase (iNOS). Bioorg Med Chem Lett 21: 2468–2471. 21398123 10.1016/j.bmcl.2011.02.061

[43] Rosenfeld RJ, Garcin ED, Panda K, Andersson G, Aberg A, et al (2002) Conformational changes in nitric oxide synthases induced by chlorzoxazone and nitroindazoles: crystallographic and computational analyses of inhibitor potency. Biochemistry (Mosc) 41: 13915–13925. 10.1021/bi026313j 12437348

[44] Liberatore GT, Jackson-Lewis V, Vukosavic S, Mandir AS, Vila M, et al (1999) Inducible nitric oxide synthase stimulates dopaminergic neurodegeneration in the MPTP model of Parkinson disease. Nat Med 5: 1403–1409. 10581083 10.1038/70978

[45] Dehmer T, Lindenau J, Haid S, Dichgans J, Schulz JB (2000) Deficiency of inducible nitric oxide synthase protects against MPTP toxicity in vivo. J Neurochem 74: 2213–2216. 10800968 10.1046/j.1471-4159.2000.0742213.x

[46] Anichtchik OV, Kaslin J, Peitsaro N, Scheinin M, Panula P (2004) Neurochemical and behavioural changes in zebrafish Danio rerio after systemic administration of 6-hydroxydopamine and 1-methyl-4-phenyl-1,2,3,6-tetrahydropyridine. J Neurochem 88: 443–453. 14690532 10.1111/j.1471-4159.2004.02190.x

[47] Lam CS, Korzh V, Strahle U (2005) Zebrafish embryos are susceptible to the dopaminergic neurotoxin MPTP. Eur J Neurosci 21: 1758–1762. 15845104 10.1111/j.1460-9568.2005.03988.x

[48] McKinley ET, Baranowski TC, Blavo DO, Cato C, Doan TN, et al (2005) Neuroprotection of MPTP-induced toxicity in zebrafish dopaminergic neurons. Brain Res Mol Brain Res 141: 128–137. 16209898 10.1016/j.molbrainres.2005.08.014

