# Supplementary figures and images for: Discovery of a Natural Product-Like iNOS Inhibitor by Molecular Docking with Potential Neuroprotective Effects In Vivo
Source: PLoS One. 2014 Apr 1;9(4):e92905. doi: 10.1371/journal.pone.0092905 (PMC3972188; doi:10.1371/journal.pone.0092905)

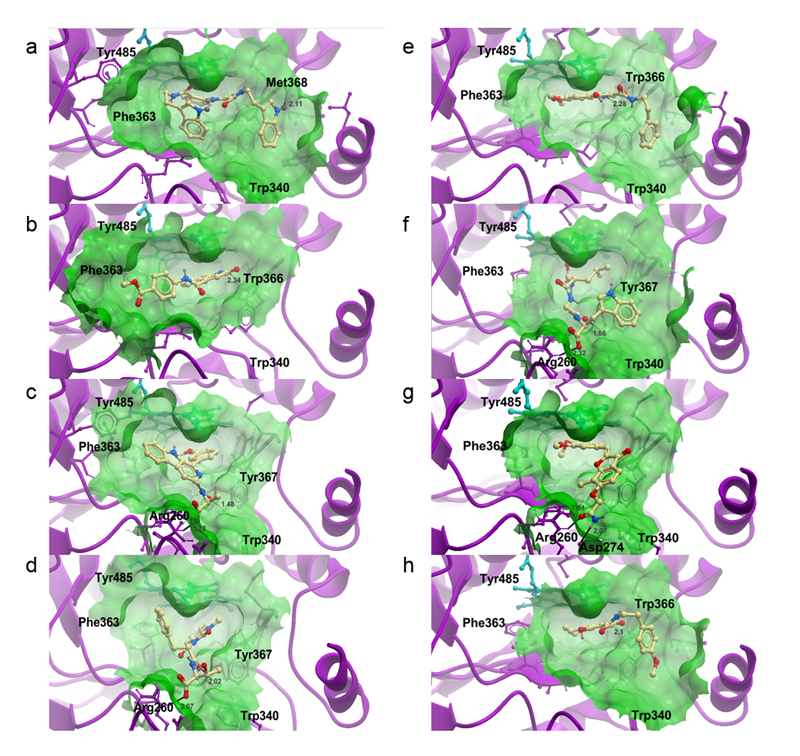

Supplement: Figure S1 — Low-energy binding conformations of compound (a–h) 1–8 bound to the iNOS molecular model by computational ligand docking. iNOS is depicted in ribbon form. The heme group and compound 1–8 are depicted as ball-and-stick models. (ZIP) [file pone.0092905.s001.zip › Figure S1.tif]
